# Linear syringocystadenoma papilliferum: an unusual presentation in the inguinal region^☆^

**DOI:** 10.1016/j.abd.2025.501224

**Published:** 2025-10-29

**Authors:** Christian Robles-Silva, Valentina Ross, Javier González, Alex Castro, Constanza Del Puerto

**Affiliations:** a Department of Dermatology, Faculty of Medicine, Universidad del Desarrollo, Clínica Alemana, Santiago, Chile; b School of Medicine, Faculty of Medicine, Pontificia Universidad Católica de Chile, Santiago, Chile; c School of Medicine, Faculty of Medicine, Universidad de Valparaíso, Viña del Mar, Chile; d Department of Pathology, Faculty of Medicine, Universidad del Desarrollo, Clínica Alemana, Santiago, Chile; e Department of Dermatology, Hospital Padre Hurtado, Santiago, Chile

Dear Editor,

Syringocystadenoma Papilliferum (SCAP) is a benign adnexal tumor characterized by apocrine differentiation.^1^ It often manifests at an early age, with approximately 50% of cases identified at birth, and 15%‒30% emerging during puberty.^2^ While most SCAPs develop de novo, up to 40% have been reported to arise from a pre-existing sebaceous nevus.^3^

The histogenesis mechanisms underlying SCAP remain unclear. Genetic studies have identified mutations in the HRAS gene in SCAPs associated with sebaceous nevi, and RAS and BRAF mutations in sporadic lesions.^1^

SCAP occurs predominantly on the head and neck region, accounting for 75% of reported cases.^3^ Other less common sites include the arms, chest, axilla, scrotum, perineal, and inguinal regions.^2^ Clinically, SCAP manifests in three forms: solitary nodule, plaque, and linear type, with the latter being extremely rare.^2^ This paper presents an unusual case of a congenital, de novo SCAP with a linear configuration located in the inguinal region.

A 29-year-old female presented with a history of several pink lesions on the right inguinal region since birth. Physical examination revealed multiple pink dome-shaped papules with central umbilication and some exhibiting surface erosions, arranged in a linear configuration (Fig. 1). Dermoscopy showed pink papillomatous structures with erosions, white rosettes, white circles, yellowish crusts, and telangiectasias (Fig. 2). Suspecting an adnexal tumor based on clinical and dermoscopic findings, an incisional biopsy was performed. Histopathological analysis revealed hyperkeratosis and acanthosis of the epidermis, with invaginations of tubular-papillary structures lined by a bilayer of cuboidal epithelium, exhibiting focal stratification and some evidence of apical secretion by decapitation. Numerous plasma cells were noted in the stroma. No evidence of cellular atypia was observed (Fig. 3).

**Fig. 1:**
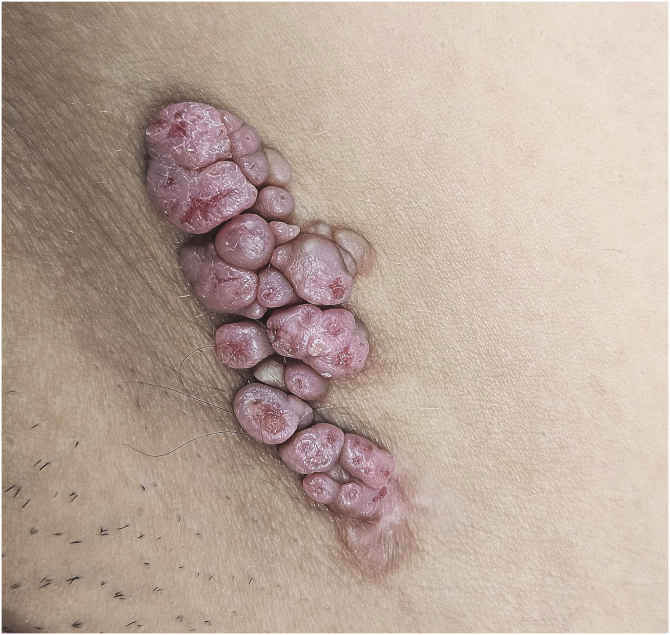
Clinical image. Multiple pink dome-shaped papules with central umbilication and some with erosions on the surface, arranged in a linear plaque.

**Fig. 2:**
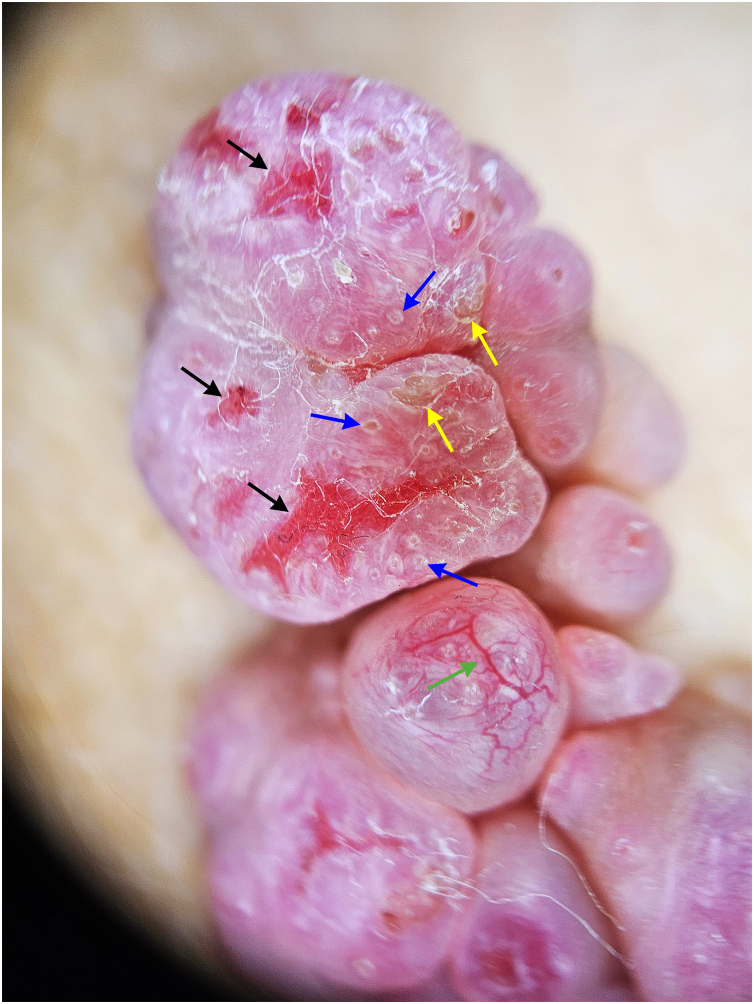
Dermoscopy image (×10): Pink papillomatous structures with erosions (black arrows), white circles (blue arrows), yellowish crusts (yellow arrows), and telangiectasias (green arrow).

**Fig. 3:**
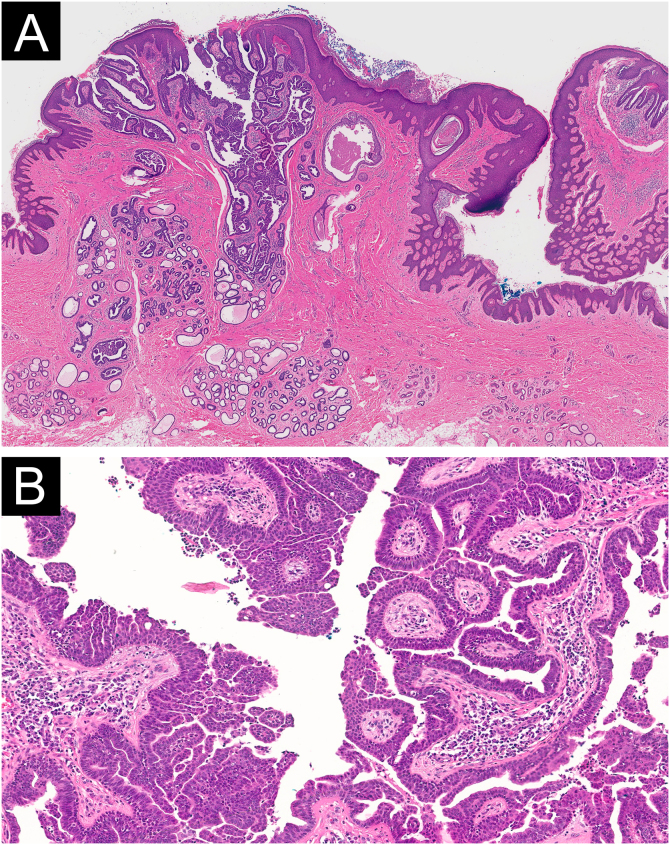
(A) Histopathological image (Hematoxylin & eosin, 20×): Epidermal and dermal tumor. The epidermis shows hyperkeratosis and acanthosis with invaginations composed of papillary projections. (B) Histopathological image (Hematoxylin & eosin, 100×): Tubulo-papillary structures lined by a bilayer cuboidal epithelium with focal stratification and some evidence of apical secretion by decapitation. The tumoral stroma contains numerous plasma cells. No cellular atypia was observed.

The diagnosis of linear syringocystadenoma papilliferum was made on a comprehensive assessment of clinical, dermoscopic, and histopathologic findings. The patient subsequently underwent a complete tumor excision, and the diagnosis was confirmed by the definitive histopathological results.

The linear presentation of SCAP is extremely infrequent, with only 22 cases reported in the literature.^2^, ^3^, ^4^ The head and neck are the most commonly affected regions, accounting for 8 of these 22 cases. Notably, only two cases have been documented in the inguinal region.^2^, ^3^

Most cases of linear SCAP are de novo occurrences. Only two cases have been associated with Jadassohn's sebaceous nevus,^2^, ^5^ two with hidrocystoma and cystadenoma,^2^, ^6^ and one with apocrine tubular adenoma.^2^

Dermoscopic findings described in SCAP include red exophytic papillary structures with central umbilication, ulceration, hairpin vessels, polymorphous vessels, and comma vessels. Additional features may include white circles, crusts, yellowish scales, and pink-white globular structures.^2^, ^3^ The circle umbilicated structures might correspond to the open pseudocystic spaces of SCAP, but further studies are needed to validate these findings.

The differential diagnosis includes molluscum contagiosum, warts, epidermal nevi, lymphangioma circumscriptum, basal cell carcinoma, and other adnexal neoplasms. Therefore, clinical and dermoscopic features are crucial techniques to identify this tumor.

Malignant transformation of SCAP is rare. Most documented cases correspond to syringocistoadenocarcinoma papilliferum,^7^ although other malignancies such as ductal carcinoma have also been reported.^8^ There are no reports of malignant transformation specifically associated with lineal SCAP.^3^

This report presents a new and exceptional case of linear SCAP located in an atypical region, with distinctive clinical and dermoscopic findings. Although the malignant transformation of linear SCAP has not been described in the literature, it is prudent to perform a complete excision. This approach is warranted as non-linear SCAP has the potential to develop secondary malignancies.

## ORCID ID

Christian Robles-Silva: 0000-0002-0263-8011

Valentina Ross: 0009-0009-3651-5536

Javier González: 0009-0008-8625-0399

Alex Castro: 0000-0003-4431-5293

Constanza Del Puerto: 0000-0003-4667-1873

## Financial support

This research did not receive any specific grant from funding agencies in the public, commercial, or not-for-profit sectors.

## Authors' contributions

Christian Robles-Silva: The study concept and design, data collection, writing of the manuscript or critical review of important intellectual content, data collection, analysis and interpretation, effective participation in the research guidance; intellectual participation in the propaedeutic and/or therapeutic conduct of the studied cases; critical review of the literature; final approval of the final version of the manuscript.

Valentina Ross: Writing of the manuscript or critical review of important intellectual content; data collection, analysis and interpretation; effective participation in the research guidance; critical review of the literature; final approval of the final version of the manuscript.

Javier González: Writing of the manuscript or critical review of important intellectual content; data collection, analysis and interpretation; effective participation in the research guidance; critical review of the literature; final approval of the final version of the manuscript.

Alex Castro: Data collection, or analysis and interpretation of data; writing of the manuscript or critical review of important intellectual content; data collection, analysis and interpretation; critical review of the literature; final approval of the final version of the manuscript.

Constanza Del Puerto: The study concept and design; data collection, or analysis and interpretation of data; writing of the manuscript or critical review of important intellectual content; data collection, analysis and interpretation; effective participation in the research guidance; intellectual participation in the propaedeutic and/or therapeutic conduct of the studied cases; critical review of the literature; final approval of the final version of the manuscript.

## Research data availability

Does not apply.

## Conflicts of interest

None declared.
